# Changes in Serum Lipid Profiles among Canine Patients Suffering from Chronic Hepatitis

**DOI:** 10.3390/vetsci8100221

**Published:** 2021-10-08

**Authors:** Sathidpak Nantasanti Assawarachan, Piyathip Chuchalermporn, Phudit Maneesaay, Naris Thengchaisri

**Affiliations:** 1 Department of Companion Animal Clinical Sciences, Faculty of Veterinary Medicine, Kasetsart University, 50 Pahonyothin Rd., Lat Yao, Chatuchak, Bangkok 10900, Thailand; sathidpak.n@ku.ac.th; 2 Endocrinology and Gastroenterology Unit, Kasetsart University Veterinary Teaching Hospital, 50 Pahonyothin Rd., Lat Yao, Chatuchak, Bangkok 10900, Thailand; 3 Radiology Unit, Kasetsart University Veterinary Teaching Hospital, 50 Pahonyothin Rd., Lat Yao, Chatuchak, Bangkok 10900, Thailand; fvetphc@ku.ac.th; 4 Department of Pathology, Faculty of Veterinary Medicine, Kasetsart University, 50 Pahonyothin Rd., Lat Yao, Chatuchak, Bangkok 10900, Thailand; fvetpdm@ku.ac.th; 5 Tippimarn Veterinary Hospital, Chulabhorn Royal Academy, 906/1 Pong Ta long Subdistrict, Pak Chong District, Nakohn Ratchasima 30130, Thailand

**Keywords:** dogs, chronic hepatitis, triglyceride, cholesterol, ultrasound score, liver enzymes

## Abstract

Hyperlipidemia is a risk factor for nonalcoholic fatty liver disease (NAFLD) in humans. However, the association between serum lipids and canine chronic hepatitis remains unknown. In this study, serum lipids, hepatic profiles, and hepatic ultrasound scores of healthy dogs and dogs with chronic hepatitis were evaluated. Serum triglyceride and cholesterol concentrations were significantly higher (*p* < 0.01) in dogs with chronic hepatitis. There were 62.2% of dogs with chronic hepatitis accompanied by hypertriglyceridemia, hypercholesterolemia, or both. Positive correlations were observed between serum ALT and cholesterol (r = 0.8287, *p* < 0.01), serum ALP and cholesterol (r = 0.8436, *p* < 0.01), serum GGT and cholesterol (r = 0.5640, *p* < 0.01), serum bile acid and cholesterol (r = 0.3310, *p* < 0.01) and serum ALP and triglycerides (r = 0.2582, *p* < 0.05). No significant differences were found between ultrasound scores of diseased dogs with and without hypertriglyceridemia and diseased dogs with and without hypercholesterolemia. Canine chronic hepatitis is associated with hyperlipidemia. A significant positive association was identified between hyperlipidemia, especially hypercholesterolemia, liver enzymes, and bile acid concentration in dogs suffering from chronic hepatitis. The underlying mechanisms connecting hyperlipidemia and canine chronic hepatitis remain elusive.

## 1. Introduction

Chronic hepatitis is a common liver disease in dogs [1], which is characterized histologically by hepatocellular apoptosis or necrosis, inflammation of hepatic parenchyma, and fibrosis. The majority of dogs with chronic hepatitis are classified as idiopathic due to the unknown pathogenesis of this disease. However, autoimmunity may play an etiological role in canine chronic hepatitis [2,3]. Dogs are diagnosed with idiopathic or immune-mediated chronic hepatitis after the elimination of infectious, metabolic, and toxicological causes, e.g., copper toxicosis [3]. Specific criteria to discriminate immune-mediated hepatitis in dogs have not been developed. The most common clinical pathology of chronic hepatitis is elevated serum alanine aminotransferase (ALT) activity, although elevated levels of serum aspartate aminotransferase (AST), alkaline phosphatase (ALP), and gamma-glutamyl transpeptidase (GGT) may be present in diseased dogs. Other abnormal hepatic function tests are hyperbilirubinemia, hypoalbuminemia, and increased serum bile acid concentration. In addition, hyperammonemia, hypocholesterolemia, and decreased blood urea nitrogen concentration are often detected in dogs with late-stage chronic hepatitis, especially cirrhosis [4]. 

Hyperlipidemia or dyslipidemia, defined as an elevation in plasma concentration of triglyceride and/or cholesterol, can be physiological (i.e., postprandial) or pathogenic (i.e., alterations in lipoprotein metabolism) [5]. The major source of cholesterol is from the diet, although this sterol can be synthesized by the liver and other tissues. The liver eliminates excess cholesterol through the plasma via low-density lipoprotein (LDL) and biliary secretion [6]. Triglycerides can also be derived from both dietary sources and hepatic synthesis [7,8]. Triglyceride hydrolysis provides fatty acids. Fatty acids can also be uptaken into the liver from the plasma and by de novo biosynthesis. Fatty acids are eliminated by β-oxidation in hepatic mitochondria or by secretion into plasma within triglyceride-rich, very-low-density lipoproteins [6]. Pathological hyperlipidemia is common in dogs and can present as a primary or secondary condition. Primary hyperlipidemia is a hereditary disorder found in miniature schnauzers, Shetland sheepdogs, Beagles, and other breeds [9,10]. Secondary hyperlipidemia can result from underlying causes such as a high-fat diet, endocrine disease, obesity, protein-losing nephropathy, cholestasis, lymphoma, or exposure to certain drugs [10]. Although hyperlipidemia itself has been considered a benign condition in dogs, it is associated with other diseases that may be potentially life-threatening, such as pancreatitis, insulin resistance induced diabetes mellitus, or seizures [10]. In humans, hyperlipidemia is associated with nonalcoholic fatty liver disease (NAFLD). Other factors such as metabolic syndromes, central obesity, hypertension, low levels of high-density lipoprotein, and hyperglycemia are also involved in disease pathogenesis [11]. Nonalcoholic steatohepatitis (NASH), an advanced type of NAFLD, is one of the most common chronic liver diseases worldwide. NASH is characterized by excessive fat deposition in hepatocytes (steatosis) and inflammation. Cirrhosis, hepatic failure, and hepatocellular carcinoma can result from NASH [12]. 

Cholestasis in dogs can result in hypertriglyceridemia and/or hypercholesterolemia [10]. Hypercholesterolemia was reported in 62.8% of dogs with cholangitis/cholangiohepatitis [13]. In addition, hypertriglyceridemia and hypercholesterolemia were observed in 66% and 93% of dogs with gall bladder mucocele, respectively [14]. Serum triglyceride and cholesterol concentrations were also significantly higher in dogs with cholelithiasis [15]. However, the concentration of serum lipids in canine chronic hepatitis has not been investigated. This study compared serum triglyceride and cholesterol concentrations between healthy controls and dogs with chronic hepatitis. We also evaluated the potential association and correlation between hyperlipidemia and levels of serum liver enzymes and bile acid. Ultrasound scores from normolipemic healthy control dogs, chronic hepatitis dogs with normolipemia, and chronic hepatitis dogs with hyperlipemia were also compared.

## 2. Materials and Methods

The use of animals in this study was approved by the Kasetsart University Institutional Animal Care and Use Committee (ACKU61-VET-012). In this study, 45 client-owned dogs diagnosed with chronic hepatitis and 45 healthy control dogs were recruited from the Kasetsart University Veterinary Teaching Hospital. All dogs were evaluated with the informed consent of their owners. Dogs with body weights of 0–12 kg, 12.01–24 kg, and > 24.01 kg were described as small, medium, and large breeds, respectively. 

Healthy control dogs did not display any clinical signs at the time of blood collection and the preceding three months. The dogs in the control group had normal complete blood counts and normal serum blood urea nitrogen; the creatinine, ALT, AST, ALP, GGT, total bilirubin, preprandial bile acid, total protein, albumin, coagulation profiles, and blood glucose were also within normal levels. The ultrasonography of the healthy dogs was performed on the same day as the blood collection. Dogs with chronic lymphoplasmacytic hepatitis were histologically confirmed by a Thai board-certified pathologist according to the WSAVA guidelines [16]. Liver samples were obtained using 14G spring-loaded needles (Argon, Frisco, TX, USA) with ultrasound guidance under generalized anaesthesia. None of the animals had been treated with corticosteroids before the start of the study. Dogs with infectious, toxic/drug-induced chronic hepatitis were excluded from this study. Dogs with concurrent diseases that cause secondary hyperlipidemia (e.g., endocrine disorders and pancreatitis) were also excluded by using clinical presentation and biochemical parameters. None of the dogs were fed a “low fat” diet or received drugs that could affect lipid metabolism (e.g., fibrate, niacin, omega-3 fatty acid) for at least three months before enrollment.

All dogs fasted for at least 12 h before the collection of blood samples. Serum triglyceride (reference interval 26–108 mg/dL) and cholesterol (reference interval 124–335 mg/dL) concentrations included ALT, AST, ALP, and GGT activities and were measured using an autoanalyzer (ILab Taurus, Milan, Italy) with the manufacturer’s reagent. Serum preprandial bile acid concentration was measured using a SNAP^®^ bile acid test (IDEXX Laboratories, Inc, Westbrook, ME, USA). Blood collection from dogs with chronic hepatitis was obtained on the same day as the liver biopsy procedure.

Ultrasonography of all lobes of the liver from healthy dogs and dogs with chronic hepatitis was performed by an experienced veterinary radiologist in a blinded fashion using a real-time scanner (GE, Fairfield, CT, USA) with a 13 MHz broadband linear transducer. All dogs fasted at least 12 h before the procedure. The ultrasound parameters and their assigned scoring system were evaluated according to the previously published literature [17]. Shortly, ultrasonographic features were categorized on the basis of (1) liver surface, (2) parenchymal score (echogenicity of parenchyma and nodularity of parenchyma), and (3) biliary score (gallbladder wall thickness, amount of gall sludge, and visibility of bile duct). Each parameter was scored with a 0, 1, 2, or 3, and the ultrasound score from each group was calculated as the sum of the scores of these parameters.

Data analysis was performed using GraphPad Prism version 6 (GraphPad Software, Inc., La Jolla, CA, USA) and STATA v.12 (StataCorp, College Station, TX, USA). All continuous variables, including triglyceride, cholesterol, ALT, ALP, GGT, and bile acid levels, were analyzed for normality. The continuous variables were evaluated using Student’s t-test. Comparisons of the associations between categorical variables, including the per cent number of animals with elevated cholesterol or triglyceride levels, was performed using the Fisher exact test. Results are expressed as mean ± standard deviation (SD). Pairwise correlation (r) analysis was conducted to determine the relationship among biochemical parameters for ALT, ALP, GGT, bile acid, triglyceride, and cholesterol. The significance level was set as *p* < 0.05.

## 3. Results

A total of 45 physically healthy dogs and 45 dogs with chronic hepatitis were evaluated. The average age of the dogs was 5.06 and 10.13 years old in the healthy group and the diseased group, respectively. The characteristics of the dogs in this study, such as sex, body weight, body condition score (BCS), and size, are summarized in Table 1. 

**Figure 1:**
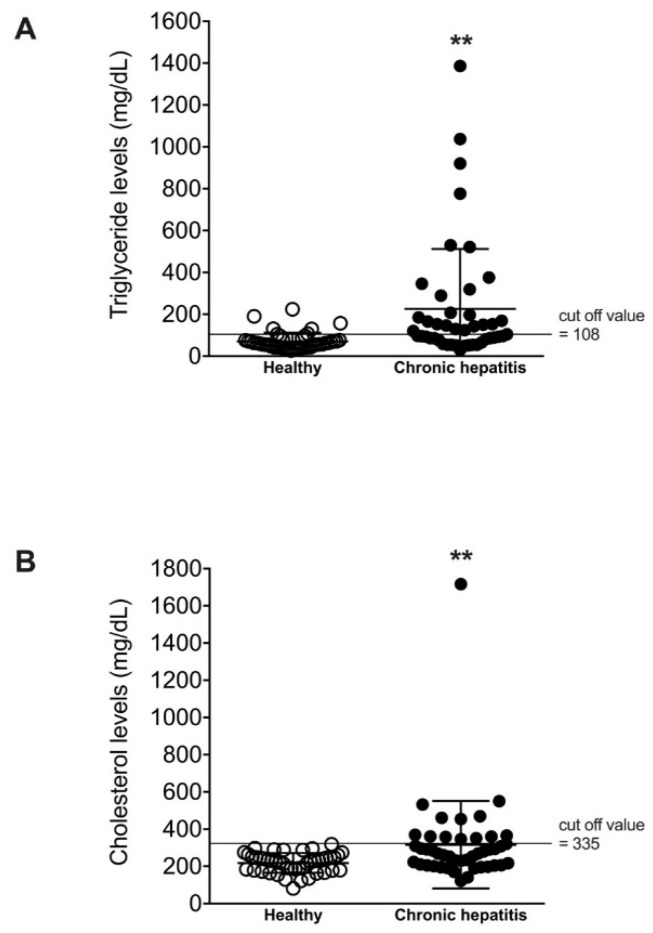
Scatterplots of serum triglyceride and cholesterol levels of healthy dogs and dogs with chronic hepatitis. Dots represent values of triglyceride levels (**A**) and cholesterol levels (**B**) for individual dogs, and lines represent mean ± SD. **p < 0.01 vs. healthy.

The serum triglyceride concentration was significantly higher (*p* < 0.01) in dogs with chronic hepatitis, 225.5 ± 286.5 mg/dL (95% CI, 139.5–311.6 mg/dL), compared to healthy dogs, 70.9 ± 40.9 mg/dL (95% CI, 58.6–83.2 mg/dL), reported as the mean ± SD (Table 1, Figure 1). In addition, the serum cholesterol concentration was significantly higher (*p* < 0.01) in dogs with chronic hepatitis, 316.3 ± 234.8 mg/dL (95% CI, 245.7–386.8 mg/dL), versus healthy dogs, 218.0 ± 52.7 mg/dL (95% CI, 202.1–233.8 mg/dL), reported as the mean ± SD. Most of the healthy dogs (88.9%, 40/45) had normal serum concentrations of triglyceride and cholesterol (Table 2). Only 11.1% (5/45) of healthy dogs had hypertriglyceridemia whereas, none of them exhibited hypercholesterolemia. In contrast, the elevation of triglyceride, cholesterol, or both triglyceride and cholesterol was found in 62.2% of dogs with chronic hepatitis. Hypertriglyceridemia and hypercholesterolemia alone were presented in 33.3% (15/45) and 11.1% (5/45) of dogs with chronic hepatitis, respectively. Interestingly, 17.8% (8/45) of dogs with chronic hepatitis had both hypertriglyceridemia and hypercholesterolemia. 

**Figure 2:**
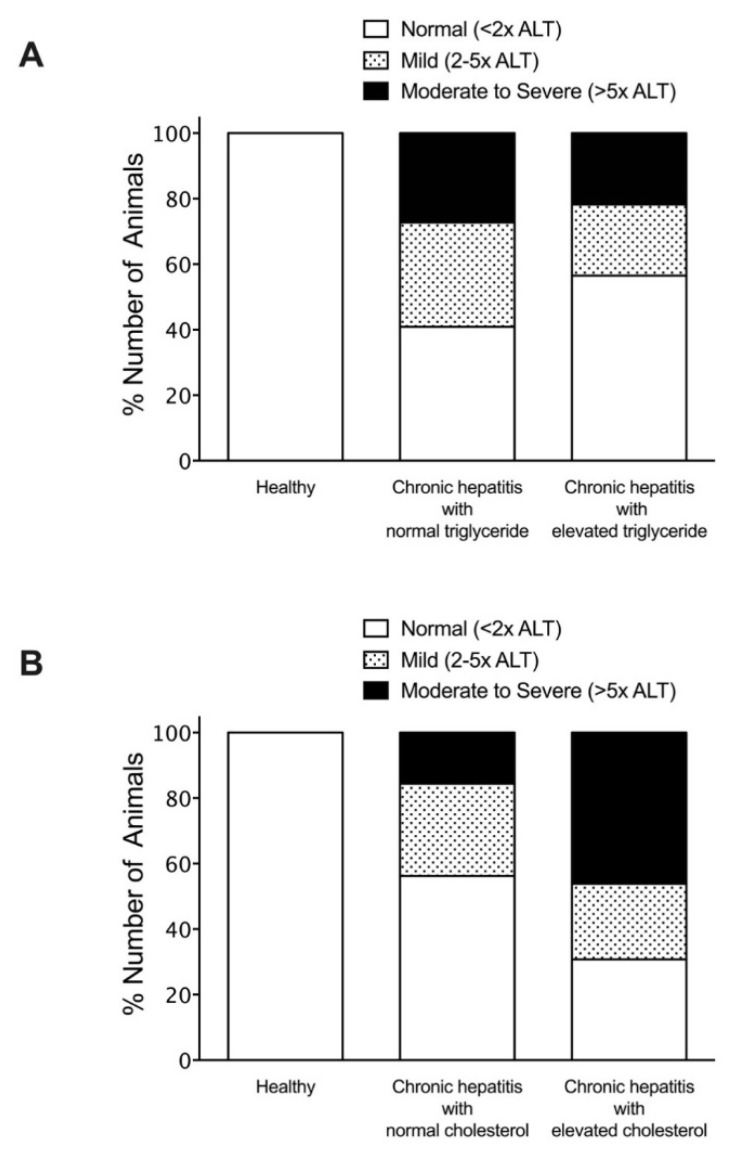
Histogram illustrating serum ALT concentrations of healthy dogs and dogs with chronic hepatitis, classified by triglyceride levels (**A**) or cholesterol levels (**B**). The magnitude of serum ALT concentration was divided into normal, mild, and moderate to severe.

**Figure 3:**
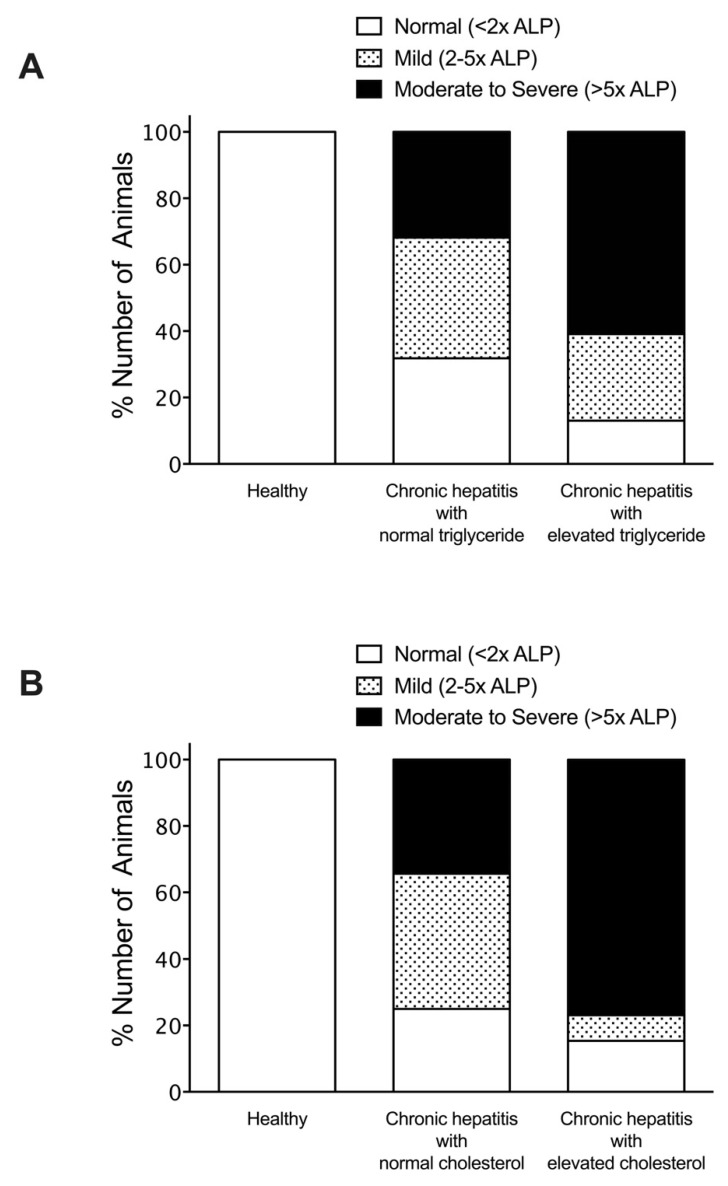
Histogram illustrating serum ALP concentrations of healthy and dogs with chronic hepatitis, classified by triglyceride levels (**A**) or cholesterol levels (**B**). The magnitude of serum ALP concentration was divided into normal, mild, and moderate to severe .

**Figure 4:**
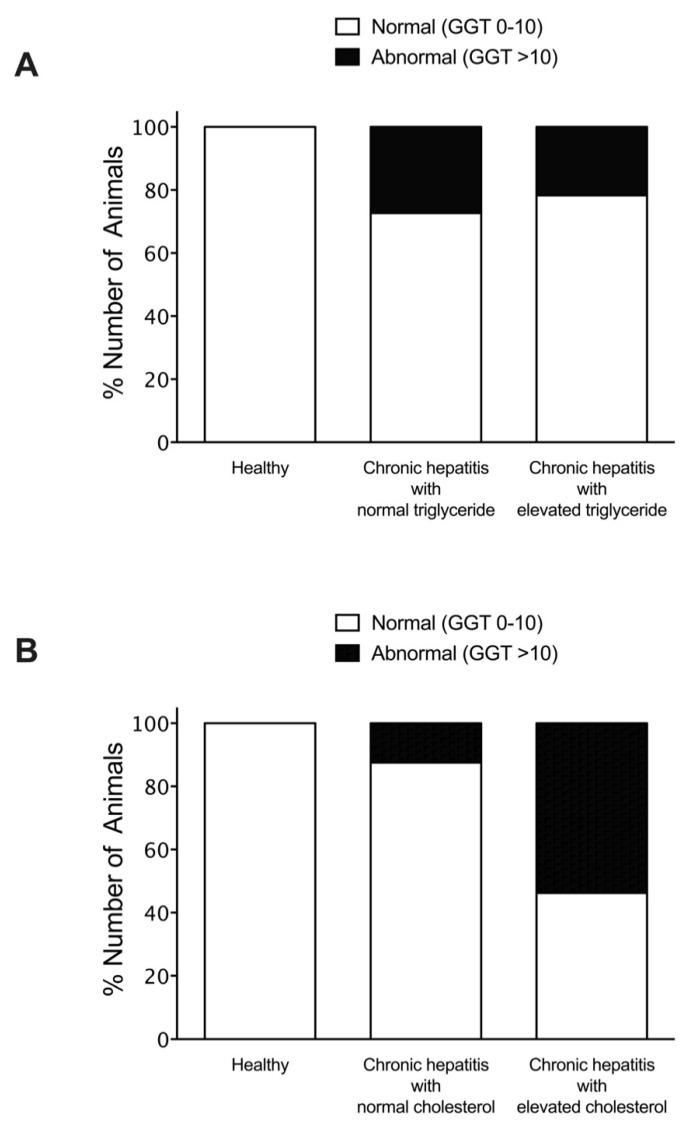
Histogram illustrating serum GGT concentrations of healthy dogs and dogs with chronic hepatitis, classified by triglyceride levels (**A**) or cholesterol levels (**B**). The magnitude of serum GGT concentration was divided into normal and abnormal.

**Figure 5:**
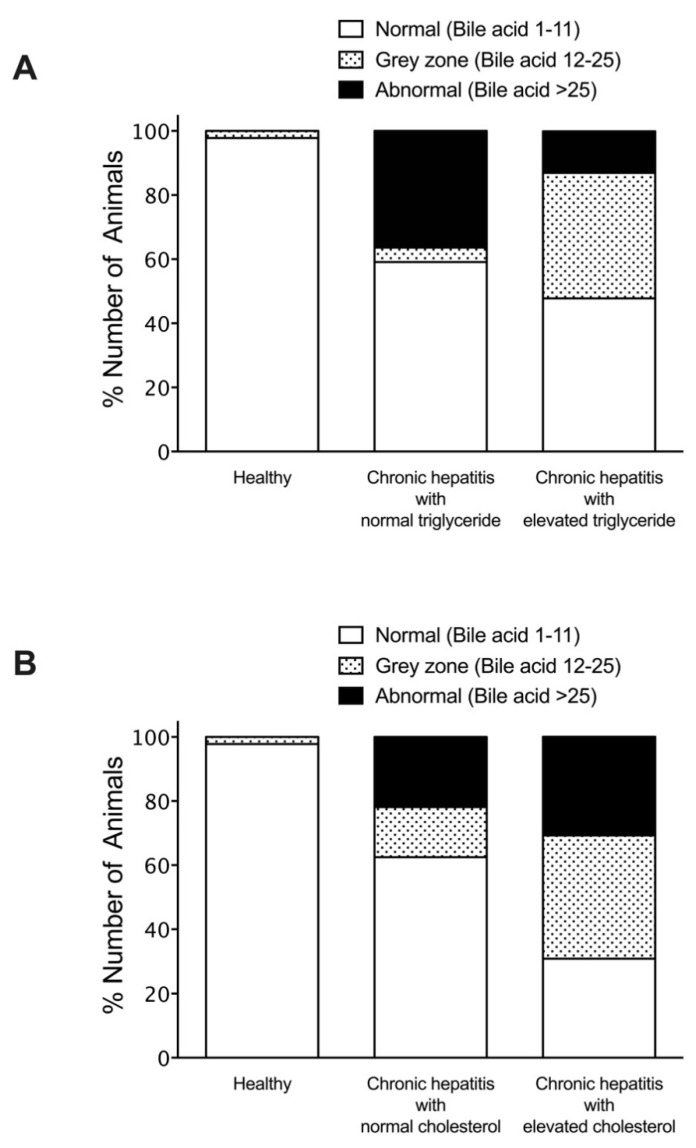
Histogram illustrating preprandial serum bile acid concentrations of healthy and dogs with chronic hepatitis, classified by triglyceride (**A**) or cholesterol levels (**B**). The magnitude of serum bile acid was divided into normal (1–11), inconclusive (grey zone) (12–25), and abnormal (> 25).

To investigate the association of hyperlipidemia on serum liver enzymes and bile acid concentrations, dogs with chronic hepatitis were classified according to the status of their triglyceride or cholesterol levels. The percentage of dogs with chronic hepatitis that had alteration of biochemical parameters included elevated ALT (60%), elevated ALP (70%), elevated GGT (25%), and elevated bile acid serum concentrations (25%). Among normotriglyceridemic diseased dogs, 27.3% exhibited moderate to severe ALT elevation, compared to 21.7% of hypertriglyceridemic diseased dogs (Figure 2). Moderate to severe ALP elevation was observed in 31.8% of normotriglyceridemic diseased dogs compared to 60.9% of hypertriglyceridemic diseased dogs (Figure 3). Abnormal GGT concentration was displayed in 27.3% of normotriglyceridemic diseased dogs compared to 21.7% of hypertriglyceridemic diseased dogs (Figure 4). Abnormal serum bile acid concentration was found in 36.4% of normotriglyceridemic diseased dogs compared to 13.0% of hypertriglyceridemic diseased dogs (Figure 5). Of the normocholesterolemic diseased dogs, 15.6% showed moderate to severe ALT elevation compared to 46.1% of hypercholesterolemic diseased dogs (Figure 2). Moderate to severe ALP elevation was found in 34.4% of normocholesterolemic diseased dogs compared to 76.9% of hypercholesterolemic diseased dogs (Figure 3). Abnormal GGT concentration was displayed in 12.5% of normotriglyceridemic diseased dogs compared to 53.8% of hypertriglyceridemic diseased dogs (Figure 4). Abnormal serum bile acid concentration was observed in 21.9% of normocholesterolemic diseased dogs compared to 30.8% of hypercholesterolemic diseased dogs (Figure 5). The results demonstrated that hypertriglyceridemia was inversely associated with the bile acid abnormality in dogs with chronic hepatitis (*p* = 0.012). Hypercholesterolemia is associated with moderate to severe serum ALP elevation and GGT elevation in dogs with chronic hepatitis (*p* = 0.03 and *p* = 0.007, respectively). 

To evaluate the relationship between hyperlipidemia, and serum liver enzymes and bile acid concentration, a Pearson correlation coefficient was performed. The results indicated that serum triglyceride concentration did not have a correlation with ALT (r = 0.0016, *p* > 0.05), GGT (r = −0.0325, *p* > 0.05), and bile acid (r = 0.0855, *p* > 0.05) concentrations; however, a low degree of correlation was found with ALP level (r = 0.2582, *p* < 0.05) (Table 3). In contrast, serum cholesterol concentration showed a high degree of correlation with ALT (r = 0.8287, *p* < 0.01) and ALP (r = 0.8436, *p* < 0.01) levels, moderate degree of correlation with GGT (r = 0.5640, *p* < 0.01), and low degree of correlation with bile acid concentrations (r = 0.3310, *p* < 0.01). 

**Figure 6:**
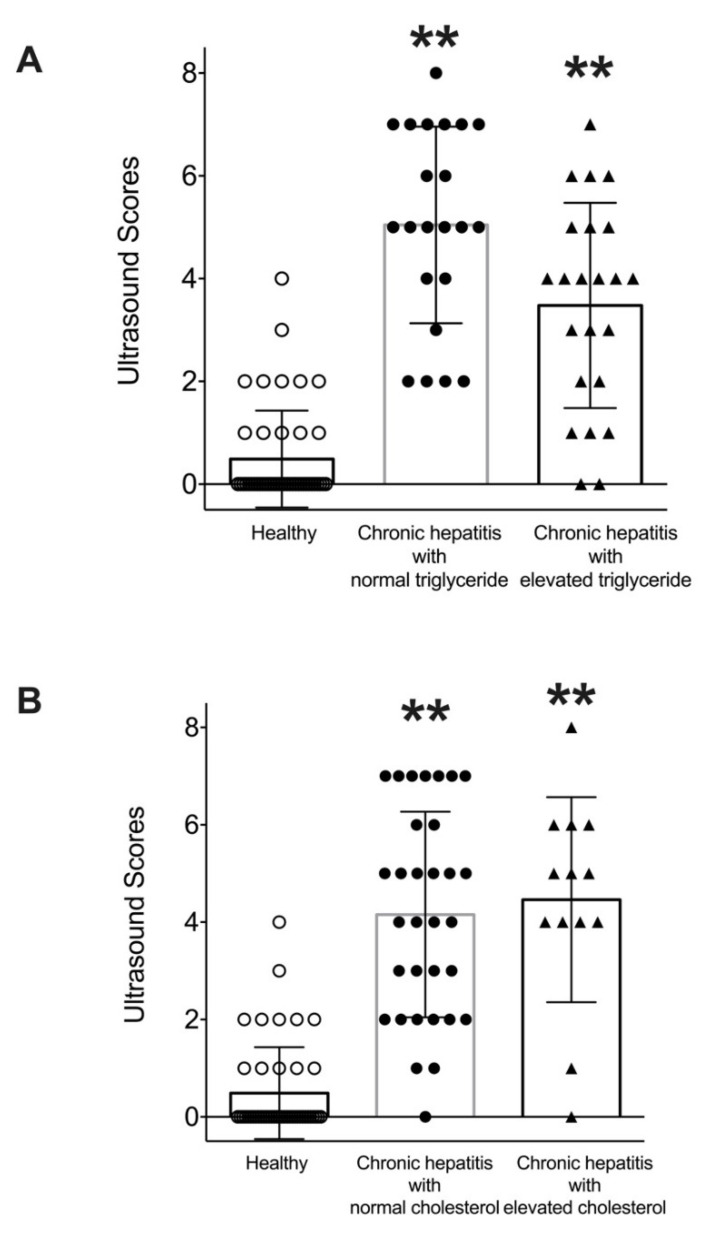
Scatterplots of ultrasound scores of healthy dogs and dogs with chronic hepatitis, classified by triglyceride (**A**) or cholesterol levels (**B**). Dots represent the ultrasound score for individual dogs, and lines represent mean ± SD. **p < 0.01 vs. healthy.

To investigate the relationship between hyperlipidemia and structural changes to the liver, ultrasonographic analysis was performed. The previously described ultrasound score [17] was determined in both groups of dogs. It was noted that diseased dogs with and without hypertriglyceridemia had higher ultrasound scores than healthy dogs (*p* < 0.01) (Figure 6). Similarly, diseased dogs with and without hypercholesterolemia had higher ultrasound scores than healthy dogs (*p* < 0.01). No significant difference was found between the ultrasound score of diseased dogs with and without hypertriglyceridemia or with and without hypercholesterolemia. 

## 4. Discussion

Our results indicate that canine chronic hepatitis is associated with hypertriglyceridemia and hypercholesterolemia. Mean values for concentrations of serum triglyceride and cholesterol were significantly higher among dogs with chronic hepatitis compared to the control group of healthy dogs. Although the average age of dogs with chronic hepatitis was higher than that of healthy dogs, a study demonstrated that plasma triglyceride and cholesterol concentration between young (0–7 years) and aged (8–13 years) dogs were not significantly different [18]. A cause-and-effect relationship between hyperlipidemia and canine chronic hepatitis has not been established. The causes of hyperlipemia in these cases may be primarily due to the livers’ central role in lipid metabolism. Hypertriglyceridemia in patients with liver disease may result from increased production of triglyceride-rich lipoproteins or decreased peripheral triglyceride catabolism due to reduction of lipase activity [19,20,21]. Damage of hepatic parenchymal cells reduced the elimination of excess cholesterol through plasma LDL and biliary secretion, possibly leading to hypercholesterolemia [6,22]. In addition, hyperlipidemia may be secondary to cholestasis, which is often found in canine chronic hepatitis [23]. Biliary excretion of cholesterol by the liver is essential to maintain normal plasma cholesterol levels [24]. Cholestasis has been reported to induce mild hypertriglyceridemia and hypercholesterolemia in both humans and dogs [10,25,26,27]. Another possible mechanism of cholestasis-induced hypercholesterolemia could be excessive esterification of lipoprotein cholesterol [28,29]. However, alterations in lipid profiles are dependent on the severity and type of liver disease. In cholestatic liver disease, cholesterol concentrations are usually elevated [30]. In contrast, cholesterol levels are lower in humans and dogs with liver diseases that reflect impaired liver synthetic function [30,31]. The dogs in this study with chronic hepatitis did not show severe hepatic dysfunction—hypocholesterolemia and other liver function test abnormalities, except increased serum bile acid, were not found. In humans, decreased serum lipid concentrations are common in patients with chronic hepatitis C infections [32]. In contrast to hepatitis c infection, serum lipid levels were not affected in human patients infected with hepatitis B. Further studies should be conducted to examine whether dogs with other types of hepatitis exhibit hyperlipidemia. 

Hypertriglyceridemia and/or hypercholesterolemia may be involved in the pathogenesis of canine chronic hepatitis, similar to that of NASH in human patients. Hypertriglyceridemia and/or hypercholesterolemia were present in 20–81% of patients with NASH. While the pathogenesis of NASH is poorly understood, it is thought to involve the accumulation of fat in hepatocytes (hepatic steatosis) due to hyperlipemia, metabolic syndrome, or other mechanisms. The increased oxidative stress and lipid peroxidation in these patients promotes hepatocyte toxicity, mitochondrial injury, and the activation of fibrinogenesis, triggering the progression to NASH, fibrosis, and cirrhosis [12,33]. Various hepatobiliary diseases in dogs are known to be associated with hyperlipidemia. To date, hypertriglyceridemia and hypercholesterolemia were shown to be risk factors for cholelithiasis and gall bladder mucocele in dogs [15,34]. Large-scale analyses are needed to identify risk factors such as lifestyle, diet types, intake of other nutrients, and toxins regarding the contribution of hyperlipidemia to the development of chronic hepatitis in dogs. Control of hyperlipidemia using lipid-lowering drugs may contribute to therapeutic utilities, amelioration of the progression of canine chronic hepatitis, or prevention of comorbidities associated with hyperlipidemia such as pancreatitis, insulin resistance, ocular disease, and seizures [10]. Measurement of lipid profiles should be regularly performed in canine patients with chronic hepatitis, as suggested by the result of the present study. 

The strong positive correlation between liver enzymes and hypercholesterolemia indicates that hypercholesterolemia may be associated with the severity of liver injury and cholestasis in canine chronic hepatitis. Serum bile acid concentration, an indicator of liver dysfunction and cholestasis [31], also showed a mild positive correlation with hypercholesterolemia. However, it should be noted that the magnitude of increased liver enzymes and bile acid does not necessarily correlate with a prognosis of liver disease [31,35]. Additional research comparing the survival time of patients with hypercholesterolemia is needed. Among all liver enzymes, only serum ALP activity was found to have a low degree positive correlation with hypertriglyceridemia. Another study in healthy miniature Schnauzers demonstrated that hypertriglyceridemia was associated with increased ALT and ALP activities [36]. Interestingly, serum bile acid concentration was inverted in association with hypertriglyceridemia. However, the Pearson correlation demonstrated no correlation between serum bile acid concentration and hypertriglyceridemia. Increased bile acid synthesis and altered bile acid distribution resulted in the reduction of serum triglyceride concentration in humans [37]. Further studies on more patients with increased serum bile acid concentration to find the association between serum bile acid and hyperlipidemia may be beneficial. 

Dogs with chronic hepatitis had higher ultrasound scores than healthy dogs, indicating significant changes to the hepatobiliary structure. However, using this ultrasound scoring system did not reveal significantly different ultrasonographic changes between diseased dogs with and without hyperlipidemia. It is possible that biochemical changes may be more sensitive than ultrasonographic changes. Moreover, hyperlipidemia found in dogs with chronic hepatitis may not cause substantial structural changes to the liver parenchyma and biliary system. Alternatively, conventional ultrasound evaluations used in this study may not be sufficiently sensitive to distinguish changes in liver structure. The use of contrast-enhanced ultrasonography or shear wave elastography may be required to properly evaluate structural changes to the liver due to hyperlipidemia. Since cholestasis may affect serum lipid levels, an association between ultrasonographic biliary abnormalities and hyperlipidemia warrants further studies.

This study has several limitations. First, liver biopsy was not performed in the healthy control dogs due to ethical issues. Clinical data, biochemical data, as well as ultrasonographic features revealed no evidence of liver lesions in healthy dogs. Moreover, we selected the younger control dogs since older dogs have a higher prevalence of liver disease [1,3,38,39,40,41]. Second, copper staining or copper quantification were not performed on the specimens in this study, as copper accumulation plays an essential role in the development of chronic hepatitis in some breeds. However, among 45 dogs with chronic hepatitis included in this study, only two dogs (West Highland White terriers and Labrador retrievers) are predisposed breeds for copper-induced hepatitis. Third, certain dog breeds might be at risk of hereditary hyperlipidemia, including Miniature Schnauzer, Shetland Sheepdogs, Beagles, and Collies [10]. In the present study, there were two Schnauzers and three Beagles with chronic hepatitis, accounting for only 11% of the population. Lastly, the present study only revealed an association between the presence of hyperlipidemia and chronic hepatitis in dogs. The underlying mechanisms of hyperlipidemia in canine patients remains unknown. Further appropriately designed studies to prove a cause-and-effect relationship between hyperlipidemia and canine chronic hepatitis is needed.

## 5. Conclusions

Canine chronic hepatitis has significantly higher serum concentrations of triglyceride and cholesterol compared to healthy control dogs. Hypercholesterolemia has a strong correlation with serum liver enzymes and bile acid. The ultrasound score was higher in dogs with chronic hepatitis than in healthy dogs; however, the score was not different between diseased dogs with and without hyperlipidemia. The underlying mechanisms linking hyperlipidemia and chronic hepatitis in canine patients remain unclear. Further studies are required to better understand the relationship between hyperlipidemia and canine chronic hepatitis. Lipid-lowering drugs may be an effective measure for improving the lipid profiles to confer more effective treatment of canine chronic hepatitis and prevent complications associated with hyperlipidemia. 

## Figures and Tables

**Table 1:** General characteristics of healthy dogs and dogs with chronic hepatitis.

Parameters	Healthy	Liver Disease
N	45	45
Bodyweight (kg, mean ± SD)	23.6 ± 16.4	12.4 ± 9.4 **
9-point BCS (mean ± SD)	6.2 ± 1.6	6.8 ± 1.8
Age (years, mean ± SD)	5.1 ± 2.8	10.1 ± 3.6 **
Sex (N)		
Male	24	21
Female	21	24
Size (N)		
Small	17	28
Medium	3	13
Large	25	4
Triglyceride (mg/dL, mean ± SD)	70.9 ± 40.9	225.5 ± 286.5 **
Cholesterol (mg/dL, mean ± SD)	218.0 ± 52.7	316.3 ± 234.8 **
ALT (IU/L, mean ± SD)	37.7 ± 12.2	361.4 ± 735.3 **
ALP (IU/L, mean ± SD)	36.0 ± 16.7	1020.5 ± 1946.3 **
GGT (IU/L, mean ± SD)	2.5 ± 1.6	14.7 ± 33.7 *
Bile acid (µmol/L, mean ± SD)	5.5 ± 2.3	13.8 ± 10.0 **

* *p* < 0.05, ** *p* < 0.01 versus healthy.

**Table 2:** Hyperlipidemia, as determined by the elevation of lipids (triglyceride > 108 mg/dL or cholesterol > 335 mg/dL), in healthy dogs and dogs with chronic hepatitis.

Hyperlipidemia	Healthy	Chronic Hepatitis
	N (%)	N (%)
Normal lipid levels	40 (88.9%)	17 (37.8%)
Hypercholesterolemia	0 (0.0%)	5 (11.1%)
Hypertriglyceridemia	5 (11.1%)	15 (33.3%)
Both hypercholesterolemia and hypertriglyceridemia	0 (0.0%)	8 (17.8%)

**Table 3:** Pairwise correlation analysis among biochemical values for ALT, ALP, GGT, bile acid, and cholesterol.

	ALT	ALP	GGT	Bile Acid	Cholesterol
Triglyceride	0.0016	0.2582 *	−0.0325	0.0855	0.2442 *
Cholesterol	0.8287 **	0.8436 **	0.5640 **	0.3310 **	-

* p < 0.05, ** p < 0.01.

## Data Availability

The datasets generated and/or analyzed during the current study are available from the corresponding author upon reasonable request.
